# Reduced soil microbial diversity and altered co-occurrence networks associated with *Phytophthora nicotianae* infection in flue-cured tobacco fields

**DOI:** 10.1128/msphere.00501-26

**Published:** 2026-08-31

**Authors:** Ayodeji Bello, Jing Yuan, Kamal Chhetri, Ran Yang, Jiayu Dong, Priyanka Gangwar, Ruizhu Shi, Zhenshan Chen, T. David Reed, Ryan Stewart, Yiming Feng, Yuan Zeng

**Affiliations:** 1 Southern Piedmont Agricultural Research and Extension Center, Virginia Tech 1757, Blackstone, Virginia, USA; 2 School of Plant and Environmental Sciences, Virginia Tech 309996 , Blacksburg, Virginia, USA; 3 College of Agricultural, Consumer, and Environmental Sciences, Las Cruces, New Mexico, USA; 4 Department of Biology, Center for Genomics and Systems Biology, New York University 118718, New York, New York, USA; 5 Virginia Seafood Agricultural Research and Extension Center, Virginia Tech 639928, Hampton, Virginia, USA; 6 Department of Biological Systems Engineering, Virginia Tech 1757, Blacksburg, Virginia, USA; 7 College of Bioscience and Resources Environment, Beijing University of Agriculture 74684, Changping District, Beijing, China; 8 Department of Agricultural and Applied Economics, Virginia Tech 1757, Blacksburg, Virginia, USA; University of Wisconsin-Madison, Madison, Wisconsin, USA

**Keywords:** soil microbial community, network patterns, *Phytophthora nicotianae*, black shank

## Abstract

*Phytophthora nicotianae* is a persistent and highly destructive soilborne oomycete that causes black shank of tobacco and continues to threaten its production despite integrated management. Although soil microbiomes are increasingly recognized as important components of disease outcomes, little is known about how soil microbial communities vary with *P. nicotianae* DNA concentrations and black shank pressure under field conditions. We examined relationships among 17 soil physicochemical properties, microbial community features, *P. nicotianae* DNA levels, and black shank outcomes in three flue-cured tobacco fields sampled repeatedly throughout one growing season. *Phytophthora nicotianae* DNA concentrations were associated with several soil physicochemical properties. The field with the lowest disease pressure had higher microbial alpha diversity, the simplest bacterial network, and a moderately complex fungal network. In contrast, the field with the highest black shank mortality showed the lowest alpha diversity, the most complex bacterial network, and the least complex fungal network. Over the growing season, microbial alpha diversity declined, and disease outcomes were associated with field-specific shifts in microbial community composition. *Trichoderma*, *Fusarium*, and *Globodera* were most abundant in the high-disease field, whereas multiple bacterial groups within Firmicutes, Proteobacteria, and Actinobacteriota were negatively associated with *P. nicotianae* in the lower-disease fields. These results indicate that *P. nicotianae* DNA abundance and black shank pressure are associated with soil physicochemical properties and field-dependent patterns in microbial diversity, community composition, network topology, and several microbial taxa. Our study provides a field-based foundation for future research to experimentally test candidate microbial indicators and microbe–pathogen relationships relevant to black shank risk assessment.

**IMPORTANCE:**

Black shank is a persistent soilborne disease that threatens tobacco production and is difficult to manage because the pathogen survives in soil and disease outcomes vary from field to field. This study shows that measuring the pathogen alone may not fully explain disease risk. Across three Virginia tobacco fields, black shank pressure was linked to soil properties and differences in the soil microbial community. Fields with lower disease pressure had higher microbial diversity and different groups of bacteria associated with lower levels of *Phytophthora nicotianae*, the causal agent of black shank. These findings suggest that future disease-risk assessment may be improved by considering soil conditions and microbial indicators together with pathogen detection. The work provides a foundation for identifying soil and microbial features that could support more targeted and sustainable black shank management strategies.

## INTRODUCTION

*Phytophthora nicotianae*, a soilborne oomycete pathogen and the causal agent of black shank disease in tobacco, poses a significant threat to tobacco production in the United States and other producing countries (1, 2). The pathogen infects tobacco at all growth stages (3), thrives in warm temperatures (23–28°C) and high soil moisture, and spreads rapidly within fields primarily through its motile zoospores. With its thick-walled chlamydospores, the pathogen can persist in soil without the presence of a host for a long period of time, making it difficult to eradicate (1, 3).

Black shank disease management mainly relies on an integrated approach that includes the use of resistant tobacco varieties carrying *Ph* genes, the *Wz* gene, or polygenic resistance loci (1, 4), along with chemical control using oomyceticides, such as metalaxyl, mefenoxam, fluopicolide, and oxathiapiprolin (5). However, repeated planting of single-gene (*Ph* or *Wz* gene) resistant tobacco varieties has resulted in *P. nicotianae* populations that overcome plant resistance (6). The use of chemical treatments also raises environmental concerns and may increase the risk of developing fungicide-resistant pathogen strains (7, 8). Soil organic amendments and cover crops, such as mustard, have been tested to manage *P. nicotianae* populations in the soil and reduce black shank severity; however, inconsistent results were found (9). Thus, there is a need to better understand the field-level biological and environmental factors associated with black shank development, including how soil microbial communities vary with *P. nicotianae* abundance and black shank disease pressure.

Over the past decade, interest has increased in developing strategies that leverage soil microbiomes to enhance crop productivity and reduce soilborne pathogen pressure (10, 11) since soil microbiota influence plant health through nutrient cycling, plant-growth promotion, competition with pathogens, and mediation of disease resistance (12). Understanding differences between microbial communities in fields with low and high disease pressure is important. In some disease-suppressive soils, microbial communities have been associated with distinct microbial taxa, higher microbial diversity, or more connected microbial networks (13, 14). However, pathogen invasion and disease development can also disrupt native soil microbial communities, leading to shifts in community structure and function that may favor disease development (15). For example, infections by *Ralstonia solanacearum* and *Verticillium dahliae* have been reported to reduce microbial diversity and enrich non-pathogenic or opportunistic organisms (16–18).

Soil structure, through its aggregation of mineral and organic matter, governs microbial community assembly by regulating oxygen, water, and nutrient availability (19, 20). In parallel, soil physicochemical properties, such as pH, organic carbon, nitrogen, moisture content, as well as agronomic factors, such as crop management history, further influence microbial metabolism, growth, and spatial distribution across successional and seasonal gradients (21–23). Variation in soil structure, physicochemical properties, and soil microbiome composition may subsequently influence soilborne pathogen activity and disease outcomes (24). Nevertheless, there remains limited understanding of how soil physical, chemical, and biological factors covary during plant disease development, hindering the identification of environmental factors associated with plant disease risk.

Despite the importance of *P. nicotianae* to tobacco production, no studies have considered how soil microbiota vary with *P. nicotianae* DNA dynamics and black shank pressure under field conditions. Clarifying these relationships may help identify microbial indicators associated with pathogen pressure and disease outcome. Here, we examined associations among soil physicochemical properties, *P. nicotianae* DNA concentrations, black shank pressure, and soil microbial diversity, composition, and co-occurrence network structure across three flue-cured tobacco fields naturally infested with *P. nicotianae*. We hypothesized that (i) microbial community diversity and composition would differ among fields and across sampling time points due to environmental heterogeneity and variations in disease pressure, and (ii) microbial co-occurrence network structure would differ among fields with different levels of black shank pressure.

## MATERIALS AND METHODS

### Experimental design

Three tobacco fields with a history of black shank disease were selected for study during the 2023 growing season. These farms were in Dinwiddie County (Barnes Farms), Brunswick County (C&C Farms), and Charlotte County (Ferrel Farms) in Virginia. Weather data from May to September 2023 were obtained from NOAA (25), with average temperatures ranging from 21.2 to 22.4°C and total precipitation of 60.9 to 64.3 cm across sites. The fields differed in farming practice, soil texture, soil map unit (https://websoilsurvey.nrcs.usda.gov), and selected pre-planting and pre-harvest soil physicochemical properties (Table 1). Agronomic practices in Barnes and C&C were conducted according to conventional practices recommended by the Virginia Cooperative Extension (26), whereas Ferrel was managed following the United States Department of Agriculture National Organic Program standards (27). At each field, three plots of 10 m × 10 m were established as replicated observational plots, with a minimum distance of 100 m between plots. No experimental treatments were applied. The flue-cured tobacco cultivar ‘NC196,’ which possesses the *Php* gene providing immunity to *P. nicotianae* race 0 and partial polygenic resistance to race 1, was used in all plots across three study fields.

**TABLE 1:** Key study site information

Parameter	Barnes	C&C	Ferrel
County	Dinwiddie	Brunswick	Charlotte
Farming practices	Conventional	Conventional	Organic
Measured soil texture	Sandy loam	Sandy loam	Sandy clay loam
USDA-NRCS soil map unit	Mattaponi sandy loam, 0–2% slopes	Emporia sandy loam, 2–8% slopes	Cecil fine gravelly sandy loam, 2–6% slopes, eroded
Flue-cured tobacco cultivar	NC 196	NC 196	NC 196
Measured pre-planting pH^*a*^	6.3 ± 0.1	6.8 ± 0.0	6.17 ± 0.15
Measured pre-planting organic matter (%)^*a*^	0.86 ± 0.06	1.53 ± 0.15	2.13 ± 0.15
Measured pre-planting inorganic matter (%)^*a*^			
Sand	78.0 ± 0.0	71.7 ± 1.5	54.3 ± 3.2
Silt	11.3 ± 1.1	15.0 ± 2.6	25 ± 4.4
Clay	9.3 ± 0.1	13.3 ± 1.5	20.7 ± 1.2
Measured pre-harvest pH^*a*^	6.6 ± 0.0	5.3 ± 0.5	6.1 ± 0.5
Measured pre-harvest organic matter (%)^*a*^	0.86 ± 0.21	1.80 ± 0.35	2.03 ± 0.06
Measured pre-harvest inorganic matter (%)^*a*^			
Sand	74.3 ± 4.6	64.3 ± 4.6	50.3 ± 1.2
Silt	13.3 ± 4.2	19.3 ± 1.2	24 ± 3.5
Clay	12.3 ± 1.5	16.3 ± 4.2	25.7 ± 4.2
Previous crop	Soybean	Soybean	Soybean
Black shank history	Yes	Yes	Yes
2023 season black shank mortality	Low	High	Intermediate

^*a*^ Values are mean ± standard deviation from pre-planting and pre-harvest soil samples. Raw data and additional soil physicochemical properties are provided in Source Data S7.

### Soil collection, sample processing, and measurements of soil physicochemical properties

Soil samples were collected monthly at each plot from pre-planting to pre-harvest. For each plot, 10 soil cores from the root-zone area (i.e., within 10 cm of each tobacco plant) were collected from a depth increment of 0–20 cm on the bed following a zig-zag pattern with a standard 2.2-cm diameter × 53 cm long soil probe (28) (AMS, American Falls, ID, USA). Soil cores from the same plot were combined, thoroughly mixed, and transported in an icebox to the Plant Pathology Laboratory at the Virginia Tech Southern Piedmont Agricultural Research and Extension Center (SPAREC) in Blackstone, VA, USA. Plant roots and debris were manually removed from soil samples before DNA extraction. A portion of each pre-planting and pre-harvest soil sample was sent to Ward Laboratories, Inc. (Kearney, NE, USA) for routine analysis to determine soil physicochemical properties, including soil pH, organic matter by loss on ignition, phosphorus by Mehlich-3 extraction, magnesium, calcium, sodium, and potassium by ammonium acetate extraction, nitrate-N by KCl extraction, sulfate by Mehlich-3 extraction, zinc, iron, manganese, and copper by DTPA extraction, sum of cations, and soil texture fractions. Principal component analysis was conducted in R (Version 4.3.2) using the prcomp function from the stats package (29) to investigate variation in pre-planting and pre-harvest soil properties among fields.

### DNA extraction, PCR amplification, and microbial sequencing

For each collected soil sample, DNA was extracted in duplicate, with each extraction performed on 0.25 g of soil using the DNeasy PowerSoil Pro Kit (Qiagen, Germany) according to the manufacturer’s protocol. The resulting DNA samples were quantified using a NanoDrop spectrophotometer (Thermo Scientific, USA). A portion of each DNA sample was sent to Novogene for bacterial 16S ribosomal RNA (16S rRNA) and fungal Internal Transcribed Spacer (ITS) library preparation and amplicon sequencing according to Novogene’s protocol. The V3–V4 regions of 16S rRNA were amplified with primers 341F (5′-CCTAYGGGRBGCASCAG-3′) and 806R (5′-GCACTACNNGGGTATCTAAT-3′) (30, 31), and ITS2 regions were amplified with ITS3-2024F (5′-GCATCGATGAAGAACGCAGC-3′) and ITS4-2409R (5′-TCCTCCGCTTATTGATATGC-3′) (32). Extraction blanks and non-template controls were included in the aforementioned polymerase chain reactions and showed no visible bands by gel electrophoresis. Amplicons were then purified with magnetic beads and quantified with Agilent 5400 Fragment Analyzer (Agilent Technologies, USA). The same amount of PCR products from each sample was end-repaired, A-tailed, and further ligated with barcodes and Illumina adapters. The quality of the resulting libraries was assessed with Qubit and the Agilent Bioanalyzer 2100 system. The quantified libraries were pooled and sequenced on Illumina NovaSeq using paired-end chemistry.

### Quantification of *P. nicotianae* DNA concentration and assessment of black shank disease severity

Soil DNA was used to estimate *P. nicotianae* DNA concentration at each sampling time point using a TaqMan-based quantitative real-time PCR (qPCR) assay following the procedure described by Li et al. (33), with modifications by comparing to an in-house prepared standard curve. Briefly, a 20 μL reaction mixture consisted of 2 μL DNA, 10 μL of 2× PrimeTime Gene Expression Master Mix (Integrated DNA Technologies) containing 0.1 μM 50 × ROX reference dye, 0.5 μL of each 10 μM forward (5′-CCTATCAAAAAAAAGGCGAACG-3′) and reverse primers (5′-TACACGGAAGGAAGAAAGTCAAG-3′), along with 0.25 μL of 10 μM probe (5′-CGGACACTGATACAGGCATACTTCCAGG-3′) labeled with FAM reporter dye and TAMRA quencher. The qPCR program included 1 cycle of initial denaturation at 95°C for 3 min, followed by 45 cycles of 95°C for 15 s and 63°C for 30 s on an Applied Biosystems QuantStudio3 Real-Time PCR System (Life Technologies, USA) to obtain cycle threshold (Ct) values. To develop a standard curve, we extracted DNA from a pure culture of *P. nicotianae* grown in 10% clarified V8 liquid media for 7 days at 28°C with constant agitation at 150 rpm. One microliter of a 10-fold serial dilution of extracted *P. nicotianae* DNA was added to 0.25 g of sterile soil, which was prepared from soil samples collected at the SPAREC tobacco black shank nursery, followed by DNA extraction. The DNA concentrations of *P. nicotianae* standard curve were 1.8 × 10^8^, 1.8 × 10^7^, 1.8 × 10^6^, 1.8 × 10^5^, 1.8 × 10^4^, and 1.8 × 10^3^ in fg/g of soil. A linear regression equation was generated by plotting Ct values against the log₁₀-transformed DNA concentrations of the standard curve. Standard curves were accepted when slopes ranged from −3.3 to −3.6, corresponding to amplification efficiencies of approximately 90–100%, and when the coefficient of determination (*R*^2^) was ≥0.98. The Ct values of tested soil samples were then applied to the linear equation to calculate *P. nicotianae* DNA concentration.

Black shank disease was assessed monthly in each plot by recording plant mortality. Plant mortality was attributed to black shank when plants showed symptoms typical of the disease, including rapid wilting, dark necrosis at the lower stem, and pith discing. When symptoms resembled bacterial wilt or Fusarium wilt, necrotic tissues were collected and brought to the lab for immunostrip testing to confirm *P. nicotianae* infection. The monthly tobacco mortality rate (%) caused by black shank disease was calculated as the number of dead plants divided by the total number of plants in each plot.


$$Motality\;rate=\left(\frac{Number\;of\;dead\;plants}{Total\;number\;of\;plants\;assessed}\right)\times 100\%$$

The area under the disease progress curve (AUDPC) was calculated using the mortality rate recorded at each time point (34) to compare the disease severity among fields.

### Bioinformatics and statistical analyses

#### Operational taxonomic units processing and quality control

The bioinformatics procedure used in this study followed the Mothur pipeline described by Zeng et al. (35), with some modifications. Briefly, contigs were created from the overlapping read pairs of each 3′ and 5′ end reads, and sequences and PCR errors were reduced, in which contigs containing ambiguous bases and longer than 450 bp for 16S rRNA or 380 bp for ITS were removed. The unique sequences were merged and aligned to the SILVA (silva.nr_v138.fasta) or UNITE ITS (UNITEv9_sh_99.fasta) databases and further de-noised to allow up to two nucleotide differences between sequences. Subsequently, chimeric sequences were detected and removed using the VSEARCH algorithm (36). The processed sequences were classified using the Bayesian classifier and clustered into operational taxonomic units (OTU) at 97% identity. Although OTUs generally provide lower sequence-level resolution than amplicon sequence variants (ASVs), OTU clustering was used to provide community-level resolution for comparing broad microbial patterns among fields and sampling times, their tolerance to sequencing errors, and downstream computational efficiency (37). OTUs with abundance sums equal to 0 were excluded after sparse abundances (counts ≤ 1) were set to 0, and the resulting matrices were used for statistical analyses.

#### Microbial diversity calculation

For alpha-diversity analysis, bacterial and fungal OTU tables were rarefied to the minimum sequencing depth within each data set using the rarefy function in the vegan package. Rarefied richness and Shannon diversity indices at the OTU level were then calculated from the rarefied OTU tables using the estimate_richness function in the phyloseq package (38). The Shannon index and rarefied richness for each soil sample were visualized using GraphPad Prism software (version 10.2.0), and generalized linear mixed models (GLMMs) in SAS OnDemand (PROC GLMM; https://welcome.oda.sas.com) were used to evaluate how they vary as functions of field, sampling time, and their interactions. When a fixed effect was significant, least squares means (LS-means) were compared using the Tukey’s adjustment (*a* = 0.05). The dissimilarities in bacterial or fungal community compositions among fields and over time were assessed using non-metric multidimensional scaling (NMDS) based on Bray–Curtis dissimilarity matrices with the vegan package (39). The effects of location, sample time, and their interaction on microbial composition were tested using permutational multivariate analysis of variance (PERMANOVA) with the adonis function in the vegan package using 9,999 permutations. The community compositions were visualized using tidyverse (40), ggplot2 (41), and ggprism (42) packages. Differences in the relative abundances of the top 10 bacterial and fungal phyla and genera among fields and sampling times were assessed using the Kruskal–Wallis test in the dplyr package (43). In addition, the top 10 bacterial and fungal genera were compared within each field and across sampling times within a field using pairwise two-sided multiple-comparison analyses with the Dwass, Steel, Critchlow-Fligner method in SAS. Prior to network construction, OTUs with a relative abundance below 0.01% of the total bacterial or fungal taxa were removed (44).

#### Co-occurrence network construction

Microbial association networks for bacterial or fungal OTUs and *P. nicotianae* were constructed using the Sparse Correlations for Compositional data (SparCC) algorithm (45) implemented from the official GitHub repository. To reduce noise, OTUs with fewer than 10 reads in each sample were removed prior to analysis. Pairwise correlations were estimated using SparCC with 200 iterations (-ni 200). To assess significance, 200 permutation data sets were generated, and empirical *P*-values were calculated. Associations among OTUs were retained if they had |*r*| ≥ 0.7 and *P* < 0.01. The resulting correlation matrix was processed in R using tidyverse, reshape2 (46), and dplyr packages to generate edge and node tables for network visualization. The network properties, such as nodes, number of edges, modularity, and average degree, were determined and visualized using the Gephi (V0.10.0) program. A lower threshold |*r*| ≥ 0.5 and *P* < 0.01 was used to include OTUs that had significant associations with *P. nicotianae* in the overall network, as previous research has reported that correlations with threshold |*r*| ≥ 0.46 and *P* < 0.01 are statistically significant when dealing with a large number of DNA samples per site (47). In the final networks, node size was scaled by the relative abundance of each OTU, node color was assigned according to phylum, and edge color differentiated positive (co-occurrence) versus negative (co-exclusion) correlations between significantly associated nodes.

#### Correlation and predictive modeling

*Phytophthora nicotianae* DNA concentrations were compared among fields and among sampling times within each field using GLMMs, with pairwise comparisons adjusted using Tukey’s method as described above. Spearman correlation analysis was conducted to determine the relationships between pre-planting soil physicochemical variables and concentration of *P. nicotianae* DNA using the rcorr function in the Hmisc package (48). To assess associations between in-season alpha diversity and *P. nicotianae*, we fit linear regressions in SAS (PROC REG) after log-transforming the pathogen variable as log(*y* + 1) to address distributional skewness. Similarly, a machine learning approach was employed to examine relationships between fungal and bacterial OTUs and *P. nicotianae* DNA concentrations under both untransformed and log-transformed scales. The OTU data were standardized using StandardScaler to ensure comparable feature scales across all measurements.

## RESULTS

### Temporal *P. nicotianae* dynamics

**Fig 1:**
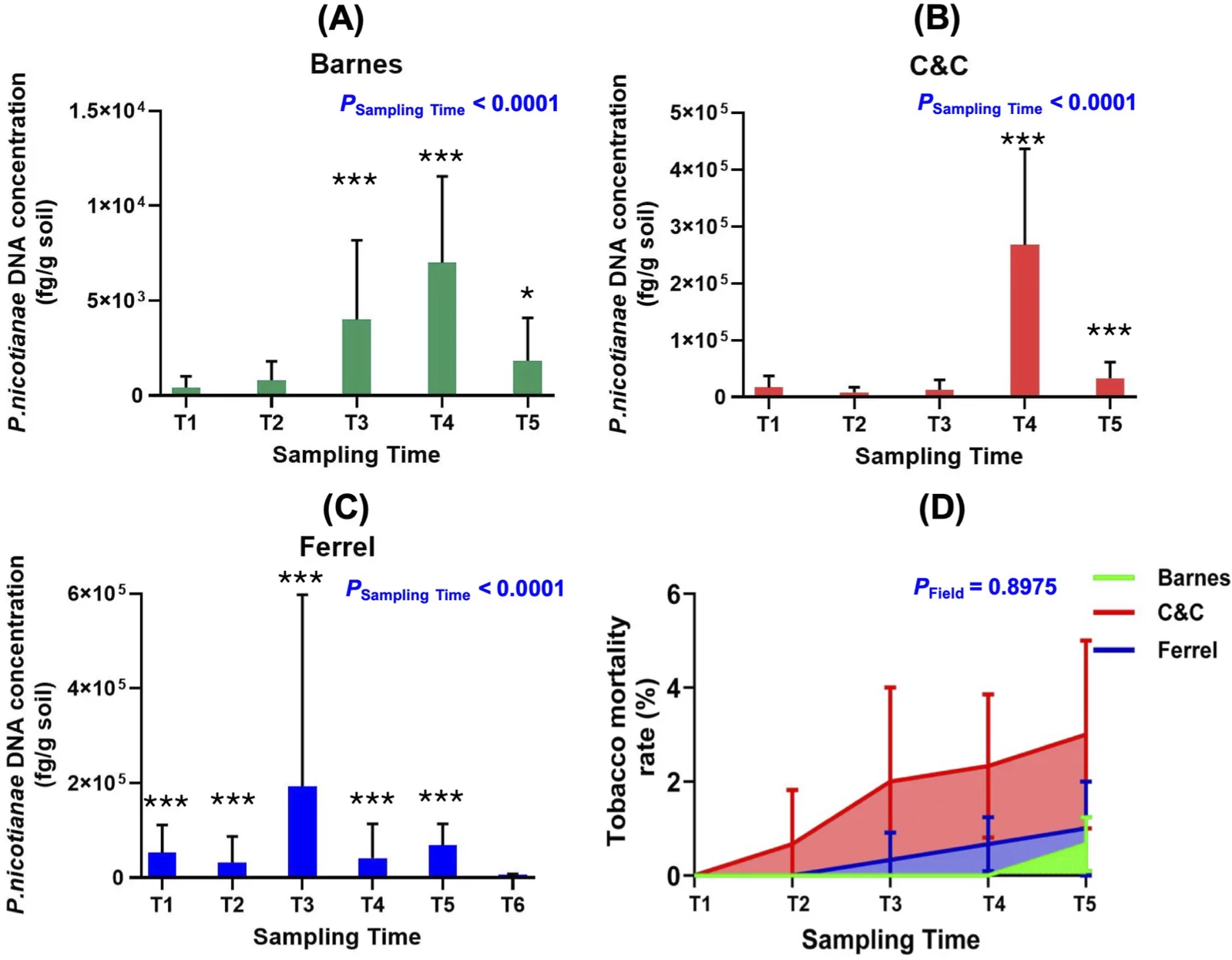
*Phytophthora nicotianae* DNA concentrations (mean + SD; fg/g of soil) in (**A**) Barnes, (**B**) C&C, and (**C**) Ferrel and (**D**) area under the disease progress curves (AUDPC; mean ± SD) of the flue-cured variety ‘NC196’ mortality rate in three flue-cured tobacco fields. *P*-values in panels **A–C** are from type III tests of fixed effects of sampling time within each field, and the *P*-value in panel **D** is from a type III test of fixed effects of field based on generalized linear mixed models. Asterisks denote a statistical difference at *a* = 0.001 (***) and 0.05 (*).

Pre-planting *P. nicotianae* DNA concentrations in soil differed among the three fields, with Barnes showing the lowest amount (346.75 ± 569 fg/g of soil), followed by C&C (1.7 × 10^4^ ± 2.0 × 10^4^) and then Ferrel (5.3 × 10^4^ ± 5.8 × 10^4^) (*P*_Field × Sampling Time_ < 0.0001; Data S1A and B). The in-season *P. nicotianae* DNA concentrations remained generally modest but displayed field-specific peaks at different sampling times (Fig. 1A through C; *P*-values_Sampling Time_ < 0.0001; Data S1A). For example, *P. nicotianae* concentration was the highest at the third sampling time (T3; 2 months after planting) in Ferrel (1.9 × 10^5^ ± 4.0 × 10^5^ fg/g of soil; *P* < 0.0001; Data S1C) when visible black shank symptoms were observed (Fig. 1C and D). At C&C, pathogen concentrations remained at similar levels until 3 months after planting (T4; 2.7 × 10^5^ ± 1.7 × 10^5^ fg/g of soil; Fig. 1B; *P* < 0.0001; Data S1C), although plant mortality was found as early as T2 (1 month after planting; Fig. 1D). At Barnes, *P. nicotianae* DNA concentrations in soil began increasing from T2 and peaked at T4, reaching 7 × 10^3^ ± 4.5 × 10^3^ fg/g of soil (Fig. 1A; *P* < 0.0001; Data S1C). However, no visible symptoms were recorded at this location until T5 (Fig. 1D). Despite dynamic variations in *P. nicotianae* concentration across fields, tobacco mortality caused by black shank disease continued to increase, with a more rapid rise observed at C&C compared to Ferrel and Barnes (Fig. 1D). However, the AUDPC scores did not differ significantly (*P* = 0.8975) among C&C (6.5 ± 1.198), Ferrel (1.5 ± 0.768), and Barnes (0.33 ± 0.289).

### Soil physicochemical factors

Of the 17 soil physicochemical parameters, 10 properties significantly correlated with pre-planting *P. nicotianae* DNA concentration (Table 2; Source Data S7). Organic matter, magnesium, calcium, the amount of nitrogen within the nitrate ion (NO_3_^−^–N), iron, manganese, and the percentage of clay and silt had a significant positive correlation with pre-planting *P. nicotianae* DNA concentrations, while sodium and the percentage of sand were negatively correlated with *P. nicotianae* concentrations. Several of the soil physicochemical variables were also strongly correlated with one another. For example, organic matter, magnesium, calcium, nitrate-N, manganese, silt, and clay were positively correlated with one another, whereas sand was negatively correlated with many of the measured variables. However, these soil physicochemical associations did not fully explain field-level disease outcomes because Ferrel had the highest pre-planting *P. nicotianae* DNA concentration but lower black shank pressure than C&C.

**TABLE 2:** Significant Spearman pairwise correlations involving pre-planting soil physicochemical properties and initial *P*. *nicotianae* DNA concentrations

Variable 1	Variable 2	Correlation coefficient	*P*-value
Soil pH	Potassium NH_4_OAc (ppm)	−0.74	0.0216
	Sulfate M-3 (ppm)	−0.82	0.0067
	Zn_DTPA (ppm)	0.87	0.0022
Organic matter	Magnesium NH_4_OAc (ppm)	0.90	0.0010
	Calcium NH_4_OAc (ppm)	0.85	0.0041
	Sodium NH_4_OAc (ppm)	−0.86	0.0032
	Nitrate-N KCl (ppm)	0.87	0.0023
	Mn-DTPA (ppm)	0.94	0.0002
	% sand	−0.94	0.0002
	% silt	0.89	0.0015
	% clay	0.91	0.0007
	*P. nicotianae*	0.93	0.0003
Phosphorus M3 (ppm)	Potassium NH_4_OAc (ppm)	−0.70	0.0358
	Sulfate M-3 (ppm)	−0.73	0.0246
	Zn-DTPA (ppm)	0.73	0.0246
Magnesium NH_4_OAc (ppm)	Calcium NH_4_OAc (ppm)	0.73	0.0262
	Sodium NH_4_OAc (ppm)	−0.70	0.0340
	Nitrate-N KCl (ppm)	0.82	0.0068
	Fe-DTPA (ppm)	0.77	0.0152
	Mn-DTPA (ppm)	0.97	0.0000
	% sand	−0.89	0.0015
	% silt	0.86	0.0030
	% clay	0.90	0.0010
	*P. nicotianae*	0.93	0.0003
Calcium NH_4_OAc (ppm)	Nitrate-N KCl (ppm)	0.87	0.0025
	Mn-DTPA (ppm)	0.78	0.0125
	Sum of cations (me/100 g)	0.78	0.0125
	% sand	−0.83	0.0056
	% silt	0.83	0.0056
	% clay	0.82	0.0074
	*P. nicotianae*	0.72	0.0298
Sodium NH_4_OAc (ppm)	Nitrate-N KCl (ppm)	−0.74	0.0218
	Sulfate M-3 (ppm)	−0.74	0.0218
	Mn-DTPA (ppm)	−0.83	0.0061
	% sand	0.87	0.0024
	% silt	−0.81	0.0079
	% slay	−0.78	0.0137
	*P. nicotianae*	−0.72	0.0302
Nitrate-N KCl (ppm)	Mn DTPA (ppm)	0.88	0.0016
	Sum of cations (me/100g)	0.67	0.0499
	% sand	−0.86	0.0026
	% silt	0.85	0.0039
	% clay	0.91	0.0007
	*P. nicotianae*	0.73	0.0246
Potassium NH_4_OAc (ppm)	Sulfate M-3 (ppm)	0.77	0.0159
	Zn-DTPA (ppm)	−0.85	0.0037
	Sum of cations (me/100g)	0.72	0.0298
Sulfate M-3 (ppm)	Zn-DTPA (ppm)	−0.92	0.0005
	Sum of cations (me/100g)	0.75	0.0199
Fe-DTPA (ppm)	Mn DTPA (ppm)	0.70	0.0358
	% sand	−0.70	0.0377
	*P. nicotianae*	0.83	0.0053
Mn-DTPA (ppm)	% sand	−0.95	0.0001
	% silt	0.93	0.0002
	% clay	0.95	0.0001
	*P. nicotianae*	0.90	0.0009
Sum of cations (me/100 g)	% sand	−0.68	0.0447
	% silt	0.73	0.0259
% sand	% silt	−0.97	< 0.0001
	% clay	−0.88	0.0017
	*P. nicotianae*	−0.88	0.0017
% silt	% clay	0.85	0.0040
	*P. nicotianae*	0.85	0.0039
% clay	*P. nicotianae*	0.79	0.0113

### Microbial diversity and community composition associated with *P. nicotianae* and black shank pressure

**Fig 2:**
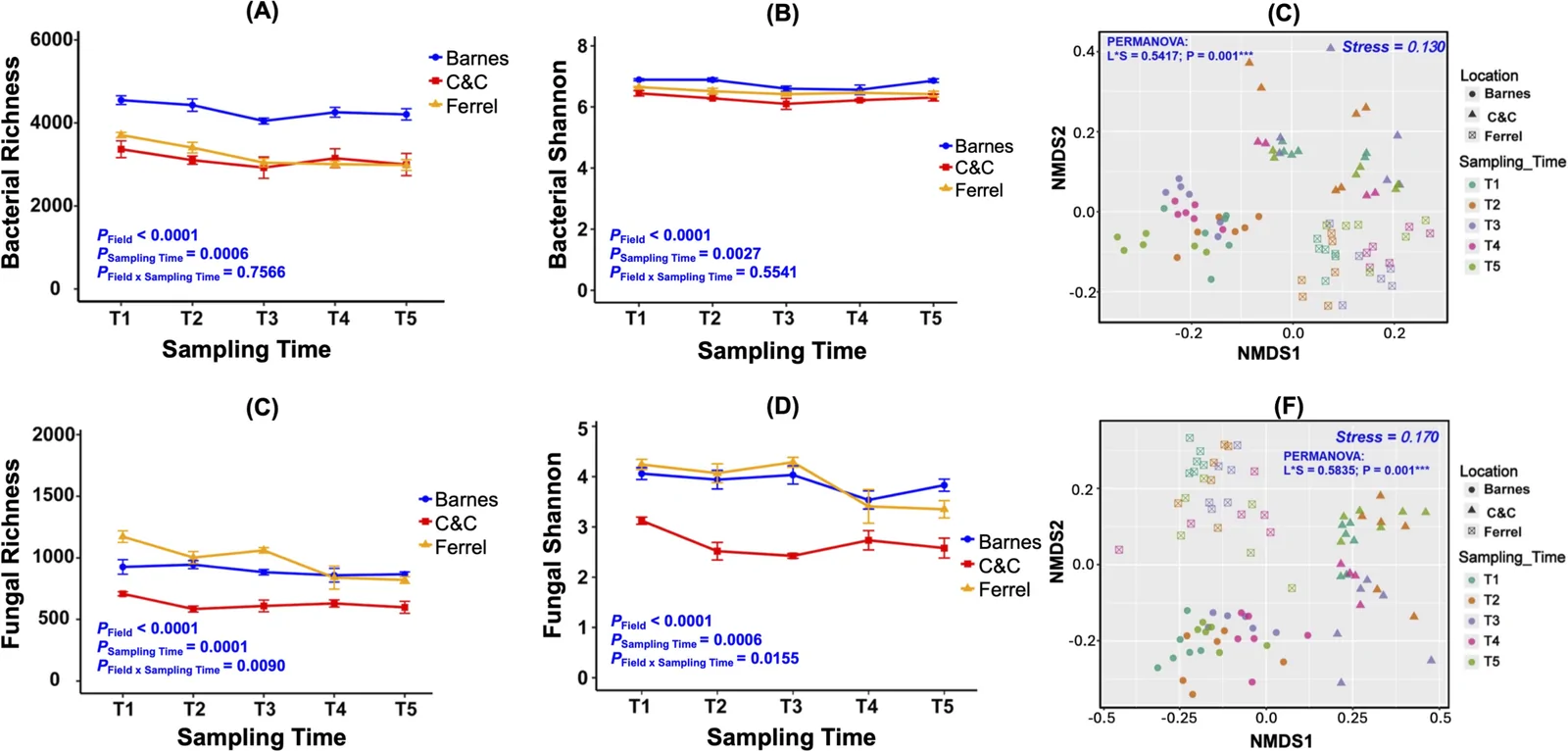
Dynamics of bacterial and fungal alpha diversity (mean ± SD) at OTU level during the tobacco growing season in Barnes, C&C, and Ferrel. (**A**) Bacterial rarefied richness and (**B**) Shannon index and (**D**) fungal rarefied richness and (**E**) Shannon index and non-metric multidimensional scaling (NMDS) plots based on Bray–Curtis dissimilarities of (**C**) bacterial community and (**F**) fungal community compositions between Barnes, C&C, and Ferrel. *P*-values shown in panels **A, B, D**, and **E** are from type III tests of fixed effects of field, sampling time, and their interactions from the generalized linear mixed model. For NMDS plots (**C and F**), stress values are shown, and *P*-values indicate differences in community composition among fields from the PERMANOVA test.

**Fig 3:**
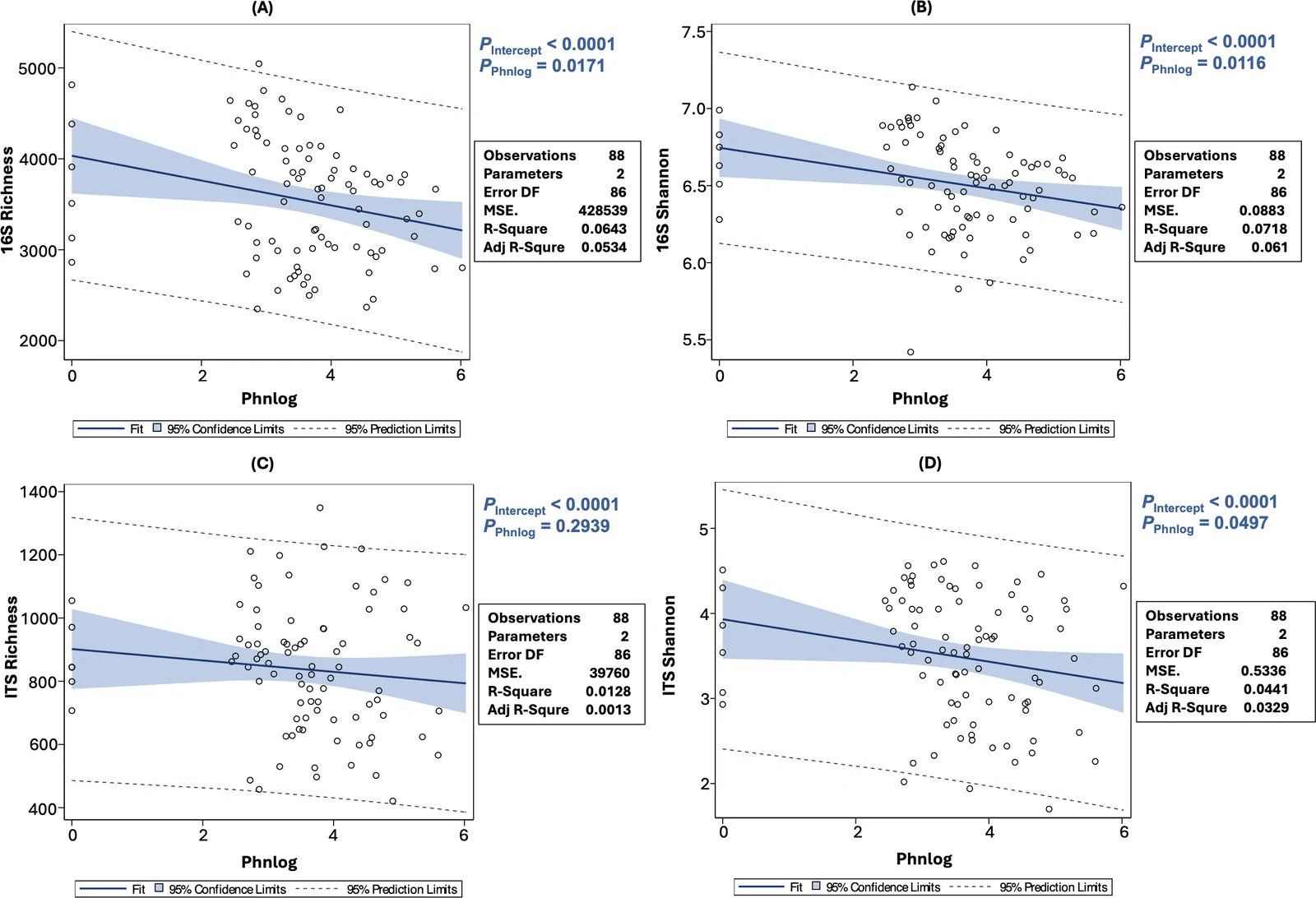
Linear relationships between log-transformed *P . nicotianae* DNA concentration [fg/g soil; log(*y* + 1)] and (**A**) bacterial richness, (**B**) bacterial Shannon diversity, (**C**) fungal richness, and (**D**) fungal Shannon diversity at OTU level.

To evaluate microbial features associated with field-level disease mortality and pathogen DNA concentration, we compared bacterial and fungal alpha diversity, community composition, and their correlations with *P. nicotianae* DNA concentrations. Rarefied richness and Shannon index of bacterial OTUs during the crop season were significantly higher in Barnes, followed by Ferrel, and then C&C (Fig. 2A and B; rarefied richness: *P*_Barnes vs. C&C_ < 0.0001; *P*_Barnes vs. Ferrel_ < 0.0001; *P*_C&C vs. Ferrel_ = 0.3362; Shannon: *P*_Barnes vs. C&C_ < 0.0001; *P*_Barnes vs. Ferrel_ < 0.0001; *P*_C&C vs. Ferrel_ = 0.0011). In contrast, fungal diversity showed a different pattern, with higher rarefied richness and Shannon diversity observed in Ferrel and Barnes compared with C&C at most sampling times (Fig. 2D and E; rarefied richness: *P*_Barnes vs. C&C_ < 0.0001; *P*_Barnes vs. Ferrel_ = 0.0217; *P*_C&C vs. Ferrel_ < 0.0001; Shannon: *P*_Barnes vs. C&C_ < 0.0001; *P*_Barnes vs. Ferrel_ = 0.9962; *P*_C&C vs. Ferrel_ < 0.0001). C&C, where the highest black shank disease mortality was recorded, had the lowest bacterial and fungal richness and Shannon diversity (Fig. 2A, B, D, and E). Across all fields, bacterial and fungal richness and Shannon diversity generally declined over the sampling period (*P-*values_Sampling Time_ < 0.01), although a transient increase in Shannon diversity was observed at the pre-harvest sampling point in Barnes. After log-transformation of *P. nicotianae* DNA concentrations, bacterial rarefied richness and Shannon diversity showed significant linear relationships with *P. nicotianae* concentrations (*P*-values < 0.05; Fig. 3A and B). Fungal Shannon diversity was also negatively associated with pathogen concentration (*P* = 0.0497; Fig. 3D), whereas fungal rarefied richness was not significantly correlated with pathogen abundance (*P* = 0.2939; Fig. 3C).

Microbial community composition differed significantly among fields based on Bray–Curtis dissimilarity (PERMANOVA: *P*_bacteria_ = 0.001; *P*_fungi_ = 0.001; Fig. 2C and F). In contrast, samples from different sampling times within the same field showed limited separation in NMDS ordination, and sampling time alone did not significantly explain community composition (Fig. 2; *P* > 0.05). However, significant field-by-sampling-time interactions were detected for both bacterial and fungal communities (*P =* 0.001), indicating that temporal community dynamics differed among fields.

The XGBoost regression models based on OTU profiles captured variation associated with *P. nicotianae* DNA concentrations, although model performance varied among train–test splits (Fig. S1). The fungal OTU-based model (Fig. S1A1 and A3) showed stronger predictive potential than the bacterial model, suggesting that fungal community composition may provide greater information for estimating *P. nicotianae* abundance in tobacco fields. However, model performance was less reliable for samples with low pathogen DNA concentrations (Fig. S1A2 and A4, Fig. S1B2 and B4).

### Field- and season-specific shifts in soil bacterial and fungal community composition

**Fig 4:**
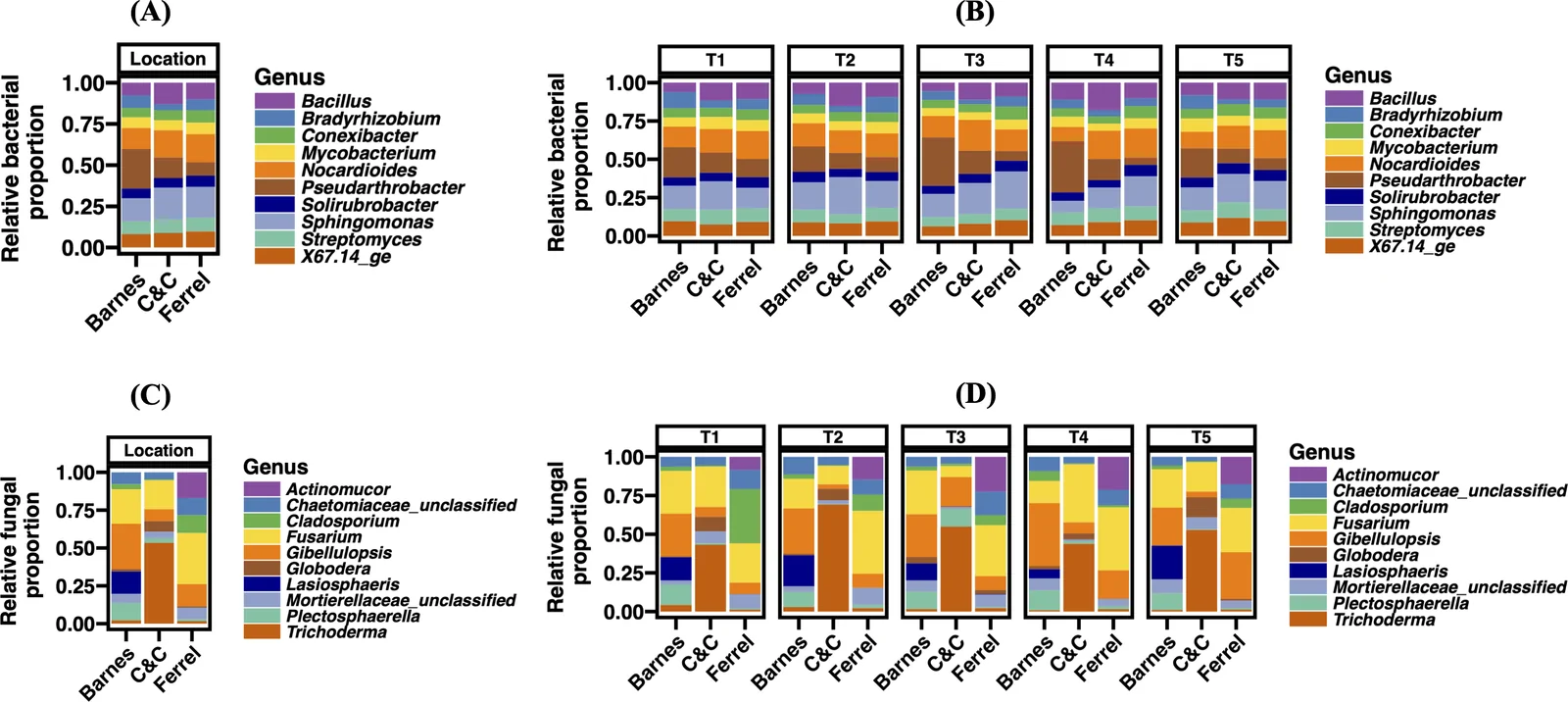
Relative proportion of the top 10 (**A**) bacterial and (**C**) fungal genera between fields and at different sampling times (**B and D**; T1–T5) at each field. Genera with less than 1% in relative abundance were not included.

The composition and relative abundance of dominant bacterial and fungal phyla were not significantly different among fields (Fig. S2; *P*-values > 0.05). Greater variation was observed at the genus level. Among the dominant bacterial genera, several groups commonly linked to plant–microbe interactions or biocontrol potential, including *Sphingomonas*, *Nocardioides*, *Pseudarthrobacter*, and *Bacillus* (Fig. 4A; Data S2 and S3), differed significantly among fields (*P <* 0.05; Table 3). Similarly, fungal community composition at the genus level varied (Table 3; *P <* 0.05), with *Trichoderma*, *Fusarium*, and *Globodera* showing field-specific patterns relevant to black shank disease mortality (Fig. 4C; Data S2 and S3).

**TABLE 3:** *P*-values for the effects of field and sampling time on the relative abundance of the top 10 bacterial and fungal genera determined by the Kruskal–Wallis test^*a*^

Genus	Field	Sampling time
Bacteria		
*Sphingomonas*	1.98 × 10^−9^***	0.03*
*Nocardioides*	4.30 × 10^−9^***	0.4949
*Pseudarthrobacter*	3.17 × 10^−9^***	0.336
*Bacillus*	4.31 × 10^−9^***	0.2442
X67.14_ge	2.04 × 10^−5^***	0.5685
*Streptomyces*	1.09 × 10^−4^***	0.0052*
*Mycobacterium*	1.35 × 10^−4^***	0.0543
*Conexibacter*	3.35 × 10^−6^***	0.3088
*Solirubrobacter*	7.98 × 10^−5^***	0.3944
*Bradyrhizobium*	0.0912	0.1073
Fungi		
*Fusarium*	1.30 × 10^−5^***	0.0739
*Trichoderma*	1.94 × 10^−13^***	0.3963
*Gibellulopsis*	1.88 × 10^−6^***	0.1777
*Chaetomiaceae_unclassified*	8.12 × 10^−7^***	0.8576
*Actinomucor*	1.95 × 10^−16^***	0.9719
*Mortierellaceae_unclassified*	0.0269*	0.0061*
*Cladosporium*	2.34 × 10^−8^***	0.809
*Plectospaerella*	2.94 × 10^−11^***	0.5758
*Lasiosphaeris*	2.08 × 10^−7^***	0.5111
*Globodera*	2.31 × 10^−15^***	0.9481

^*a*^ Asterisks denote a statistical difference at *a* = 0.001 (***) and 0.05 (*).

Temporal variation among dominant genera was limited, with only *Sphingomonas*, *Streptomyces*, and *Mortierellaceae_unclassified* varying significantly between sampling times (Table 3). Because C&C had the highest black shank mortality, we further examined temporal patterns of dominant fungal genera in this field. From T3 to T4, the relative abundance of *Fusarium* and *Globodera* increased concurrently with *P. nicotianae* concentrations in C&C soils [Fig. 4D; Fig. S3B; *R*^2^_(*Fusarium-Phn*)T3:T4_ = 0.6632; *P* = 0.0366; *R*^2^_(*Globodera-Phn*)T3:T4_ = 0.0876; *P* = 0.8098]. The increase was significant for *Fusarium* (*P_Fusarium_*_:T3vs.T4_ = 0.0366) but not for *Globodera* (*P_Globodera_*_:T3vs.T4_ = 0.4304; Data S4).

### Soil microbial networks associated with *P. nicotianae* and black shank disease mortality

**Fig 5:**
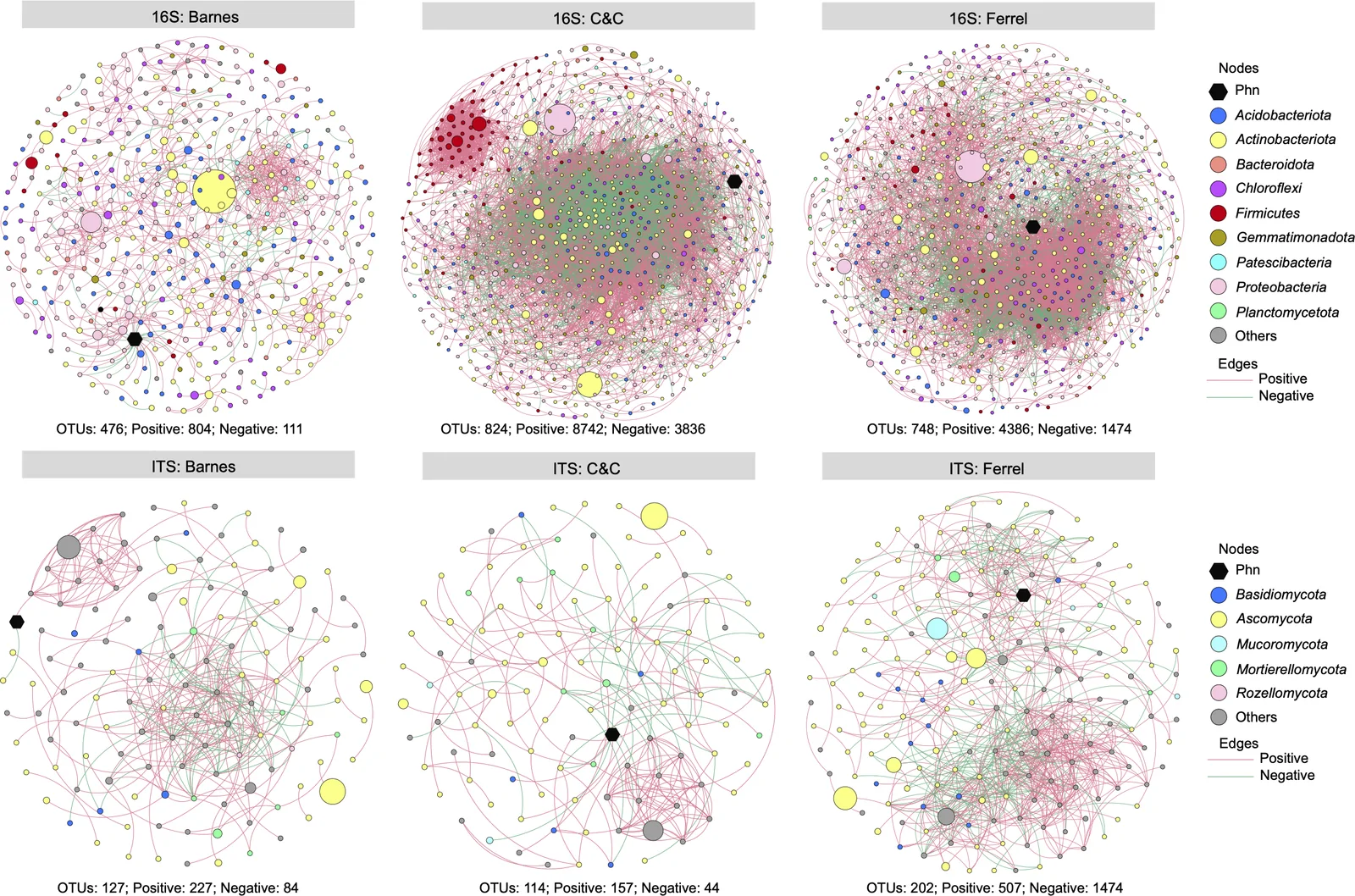
SparCC-based co-occurrence networks of soil bacterial (16S) and fungal (ITS) communities by field. Nodes represent OTUs colored by phylum, and node size is proportional to the relative abundance of each OTU. Edges indicate significant correlations between two OTUs or between *P. nicotianae* and an OTU (positive correlations: co-occurrence; negative correlations: mutual exclusion; |*r*| ≥ 0.7, *P* < 0.01).

Microbial co-occurrence network topology differed among fields, with distinct patterns observed between bacterial and fungal communities (Fig. 5). The bacterial network in C&C exhibited greater connectivity than those in Barnes and Ferrel, characterized by higher numbers of nodes and edges, average degree, and graph density (Table 4; |*r*| ≥ 0.7, *P* < 0.01). In contrast, the fungal network in C&C showed reduced connectivity related to Barnes and Ferrel (Fig. 5). These results indicate field-specific differences in microbial association patterns, with contrasting responses between bacterial and fungal communities.

**TABLE 4:** Major bacterial and fungal network features (|*r*| ≥ 0.7, *P* < 0.01) in each field

Network properties	Bacteria			Fungi		
	Barnes	C&C	Ferrel	Barnes	C&C	Ferrel
Nodes	476	824	748	127	114	202
Edges	915	12,578	5,850	311	201	738
Positive edges	804	8,742	4,376	227	157	507
Negative edges	111	3,842	1,474	84	44	231
Average degree^*a*^	1.918	15.246	9.101	2.43	1.748	3.635
Network diameter^*b*^	7	10	10	5	4	8
Graph density^*c*^	0.004	0.019	0.010	0.019	0.015	0.018
Modularity^*d*^	0.8031	0.345	0.387	0.58	0.741	0.557
Average clustering coefficient^*e*^	0.128	0.236	0.221	0.187	0.152	0.199
Average path length^*f*^	2.391	3.003	2.706	1.902	1.392	2.656

^*a*^ Average number of edges per node in the network.

^*b*^ Longest shortest path between any two nodes in a network.

^*c*^ Measure of how many edges in a graph relative to the maximum possible number of edges.

^*d*^ Estimate of the strength of the division of a network into communities or clusters.

^*e*^ Tendency of nodes in a network to cluster together.

^*f*^ Number of edges in the shortest path between two nodes.

To identify microbial taxa associated with the black shank pathogen dynamics, OTUs significantly correlated with *P. nicotianae* were extracted from each network (|*r*| ≥ 0.5 and *P* < 0.01; Data S5). Several bacterial taxa showed field-specific negative associations with *P. nicotianae*. In Barnes and Ferrel, negatively correlated bacterial OTUs were predominantly affiliated with Firmicutes and included *Cohnella*, *Paenibacillus*, *Clostridium_sensu_stricto_8*, *Bacillus*, *Tumebacillus*, *Bacillaceae_unclassified*, and *Rummeliibacillus*. These taxa were not detected as pathogen-associated network members in C&C, the field with the highest black shank mortality (Table 5; Data S5).

**TABLE 5:** Number of taxa at the phylum level significantly correlated with *P. nicotianae^a^*

Kingdom	Phylum	Negative correlation			Positive correlation		
		Barnes	C&C	Ferrel	Barnes	C&C	Ferrel
Bacteria	Actinobacteriota	1	0	5	6	1	5
	Acidobacteriota	3	2	1	3	0	5
	Bacteroidota	1	0	0	0	0	0
	Chloroflexi	1	0	1	0	3	6
	Firmicutes	3	0	4	0	1	0
	Gemmatimonadota	0	2	1	1	0	2
	Methylomirabilota	0	0	0	0	0	1
	Myxococcota	0	0	0	0	0	2
	Proteobacteria	10	2	6	1	2	6
	RCP2-54	0	0	0	0	0	1
Fungi	Ascomycota	1	3	6	0	1	6
	Basidiomycota	0	1	0	0	1	0
	Fungi_unclassified	0	0	3	1	0	0
	Mucoromycota	0	1	0	0	0	0

^*a*^ Note: |*r*| ≥ 0.5, *P* < 0.01.

Ferrel, which exhibited the lowest disease mortality despite having the highest initial *P. nicotianae* DNA abundance (Fig. 1), contained several unique bacterial taxa negatively associated with *P. nicotianae.* These included five Actinobacteriota taxa, including *Jatrophihabitans*, *Geodermatophilus*, *Crossiella*, *Acidimicrobiaceae_uncultured*, and *Gaiella* (Data S5). Similarly, more Proteobacteria taxa were negatively correlated with *P. nicotianae* in Barnes and Ferrel than in C&C.

Fungal taxa associated with *P. nicotianae* were primarily detected in C&C. Specifically, *Mucor* (Mucoromycota) exhibited a negative association, whereas *Vishniacozyma* and *Phallus* (Basidiomycota) showed positive associations with *P. nicotianae* (Data S5).

## DISCUSSION

### Black shank development reflects interactions among pathogen abundance, soil physicochemical properties, and microbial communities

In-season *P. nicotianae* DNA concentrations varied strongly over time and peaked at different sampling points. However, these dynamics did not correspond consistently with tobacco mortality and AUDPC scores, indicating that black shank development in field settings is not determined by pathogen DNA abundance alone. Rather, it is likely shaped by interactions among pathogen presence, host condition, soil physicochemical properties, weather, and microbial communities (49–53). The positive associations between *P. nicotianae* DNA concentrations and organic matter, calcium, iron, magnesium, manganese, nitrate-N, and percentages of silt and clay suggest that pathogen DNA was greater in soils with higher nutrient availability and finer-textured fractions. Finer-textured soils generally have greater water-holding capacity and cation retention than sandier soils, which may create conditions that support pathogen persistence or detectable pathogen DNA under favorable moisture conditions (54). Although the sand percentage was negatively associated with *P. nicotianae* DNA concentration in this study, soil texture effects on pathogen movement are likely context-dependent (55).

Soil microbial alpha diversities differed significantly among fields and declined throughout the cropping season, with a non-significant late-season rebound in Barnes, indicating that higher pathogen loads or associated disease pressure coincide with reduced microbial alpha diversity and that these relationships may reflect enrichments in specific microbial groups that respond to disease-affected roots, including taxa within Proteobacteria and Actinobacteria (56–58). In contrast, microbial community composition was primarily structured by field, as supported by both NMDS ordinations and PERMANOVA tests. Although within-field differences among sampling times were not visually distinct, PERMANOVA detected a significant location and sampling time interaction for both bacterial and fungal communities, indicating that seasonal changes in community composition were field dependent. The association between microbial community composition and *P. nicotianae* DNA concentration further suggests that bacterial and fungal OTU profiles may contain pathogen-associated signals in field soils. Overall, these results suggest that each field maintained a distinct microbial community structure shaped largely by local soil characteristics, while still undergoing field-specific seasonal shifts that may have been influenced in part by pathogen activity and disease occurrence.

### Field-specific microbial taxa show associations with black shank pressure and *P. nicotianae* abundance

Several bacterial genera commonly linked to plant–microbe interactions and biocontrol potential, including *Sphingomonas*, *Nocardioides*, *Pseudarthrobacter*, *Bacillus*, and *Streptomyces,* showed significant differences among fields and, for a subset of taxa, at specific sampling timepoints. The abundance of *Trichoderma* also varied dramatically among fields, with the highest levels observed at C&C, the field with the highest black shank mortality. This difference may be attributed, in part, to soil physicochemical properties, such as the more neutral pH at C&C, that favor *Trichoderma* establishment (59). Within C&C, *Fusarium* increased from T3 to T4 alongside *P. nicotianae* concentrations and showed a significant positive association over that interval, whereas the corresponding association for *Globodera* was not significant. Because *Fusarium* includes important tobacco root pathogens (e.g., *Fusarium oxysporum*, *F. solani*, and *F. annulatum*) (60), and *Globodera* comprises plant-parasitic nematodes (61), the co-variation observed for *Fusarium* during peak *P. nicotianae* abundance is notable. However, metagenomic sequencing and targeted isolation will be needed to classify the taxa involved and test mechanisms underlying their co-occurrence with *P. nicotianae* under high disease pressure.

### Microbial network complexity does not necessarily indicate disease suppression

Network analysis further highlighted field-specific differences in microbial co-occurrence structure associated with *P. nicotianae*. Previous studies suggested that complex and highly connected soil microbial networks are often associated with disease-suppressive soils (13, 62). However, using a stringent SparCC threshold (|*r*| ≥ 0.7, *P* < 0.01), bacterial networks were most complex at C&C and least complex at Barnes, contrary to our second hypothesis. This result indicates that greater microbial network complexity does not necessarily correspond to lower disease pressure and, in this system, may instead reflect shifts in bacterial co-occurrence associated with disease-affected roots. In Barnes, where disease pressure was low, bacterial networks showed lower overall complexity but a higher number of major modules, consistent with a more compartmentalized community structure that may be more resilient to disturbance (16, 62).

### Microbial taxa negatively associated with *P. nicotianae* represent candidates for functional validation

Many Firmicutes, especially *Bacillus* and *Paenibacillus*, are known to produce antimicrobial metabolites, lytic enzymes, and compounds that can induce host defenses (63–65). Their exclusive occurrence among OTUs negatively associated with *P. nicotianae* in the lower-disease fields (Barnes and Ferrel) is consistent with a potential role for *Firmicutes* in pathogen suppression or competitive exclusion. Similarly, the greater number of *Proteobacteria* negatively associated with *P. nicotianae* in Barnes and Ferrel suggests that competitive interactions and biofilm formation may be relevant to pathogen-associated community structure (66, 67). Ferrel also harbored unique *Actinobacteriota* OTUs that were negatively associated with *P. nicotianae*, which may be relevant because many *Actinobacteria* include taxa capable of producing secondary metabolites frequently implicated in disease-suppressive soils (68). Although SparCC-based networks can identify field-specific associations in compositional microbiome data, they do not demonstrate direct microbial interactions, antagonism, or causal effects on disease development. Therefore, taxa negatively associated with *P. nicotianae* should be considered microbial candidates for future isolation, genomic, metabolomic, and functional assays rather than confirmed suppressive organisms.

In this study, we included replicated plots within each field and repeated sampling in the growing season, which allowed us to detect field-specific microbial and pathogen-associated patterns. However, repeating the same field experiment across consecutive years was not feasible because cooperating growers rotate tobacco with other crops, limiting access to the same tobacco fields and disease conditions in subsequent years. Therefore, future studies across additional tobacco production seasons and sites could further evaluate the broader applicability of the microbial patterns identified here.

### Conclusions

This study improves our understanding of how soil physicochemical properties and microbial community structure are associated with *P. nicotianae* abundance and black shank disease mortality in flue-cured tobacco fields. Notably, the field with the lowest disease pressure had higher microbial alpha diversity, the simplest bacterial network, and a moderately complex fungal network, whereas the field with the highest black shank mortality had the lowest alpha-diversity, the most complex bacterial network, and the least complex fungal network. These field-level patterns suggest that microbial community features co-vary with disease pressure in ways that are not explained by pathogen inoculum levels alone. More broadly, our study suggests that black shank risk assessment may benefit from integrating pathogen detection with soil physicochemical and microbiome information, including candidate microbial indicators negatively associated with *P. nicotianae*. Future studies across additional fields, seasons, and production systems will be needed to test the broader applicability of these soil and biological indicators and their relevance to microbiome-informed disease management.

## Data Availability

The raw sequence data were deposited in the National Center for Biotechnology Information (NCBI) Sequence Read Archive with accession numbers PRJNA1250572 for bacteria and PRJNA1250608 for fungi.

## SUPPLEMENTAL MATERIAL

Supplemental Figures msphere.00501-26-s0001.pdf Fig. S1 to S3.

Source Data msphere.00501-26-s0002.xlsx Source Data S1 to S8.

Supplemental Data msphere.00501-26-s0003.xlsx Data S1 to S5.

ASM does not own the copyrights to Supplemental Material that may be linked to, or accessed through, an article. The authors have granted ASM a non-exclusive, world-wide license to publish the Supplemental Material files. Please contact the corresponding author directly for reuse.

## References

[1] Gallup CA, McCorkle KL, Ivors KL, Shew D. 2018. Characterization of the black shank pathogen, *Phytophthora nicotianae*, across North Carolina tobacco production areas. Plant Dis 102:1108–1114. doi:10.1094/PDIS-02-17-0295-RE 30673436

[2] Zhu XQ, Chen Y, Jia M, Dai HJ, Zhou YB, Yang HW, Zhou P, Du Y, Wang G, Bai YX, Wang N. 2024. Managing tobacco black shank disease using biochar: direct toxicity and indirect ecological mechanisms. Microbiol Spectr 12:e00149-24. doi:10.1128/spectrum.00149-24 PMC11448098 39212424

[3] Cochran S, Quesada-Ocampo LM, Kerns JP, Thiessen LD. 2024. *Phytophthora nicotianae*: a quick diagnostic guide for black shank of tobacco. Plant Health Prog 25:327–333. doi:10.1094/PHP-10-23-0085-DG

[4] Zeng JM, Nifong J, Liu Y, Huang CJ, Fang DH, Lewis RS, Li YP. 2019. Evaluating diverse systems of tobacco genetic resistance to *Phytophthora nicotianae* in Yunnan, China. Plant Pathol 68:1616–1623. doi:10.1111/ppa.13091

[5] Webb AB, Bailey WA. 2024. Chemical control of black shank (*Phytophthora nicotianae*) in strip-tillage dark fire-cured tobacco. Tob Sci 58:9–12. doi:10.3381/TOBSCI-D-23-00001

[6] Jin J, McCorkle KL, Cornish V, Carbone I, Lewis RS, Shew HD. 2022. Adaptation of *Phytophthora nicotianae* to multiple sources of partial resistance in tobacco. Plant Dis 106:906–917. doi:10.1094/PDIS-06-21-1241-RE 34735283

[7] Aguiar TR Jr, Bortolozo FR, Rosa Filho EF, Parron LM, Luz LD, Brito AG, Ferreira MT. 2017. Fate of selected agrochemicals in a tropical karst aquifer: a five-year study. Groundw Sustain Dev 5:187–192. doi:10.1016/j.gsd.2017.06.008

[8] Chatrath MS, Raychaudhuri SP, Gupta DS. 1980. Fungicides as an environmental problem: studies on translocation and persistence in host and soil, p 273–280. *In* Environmental pollution & toxicology: international symposium

[9] Holdcroft AM. 2013. Alternative methods of control for *Phytophthora nicotianae* of tobacco. MS thesis, University of Kentucky, Lexington, KY. https://uknowledge.uky.edu/plantpath_etds/5.

[10] Ahkami AH, Allen White R III, Handakumbura PP, Jansson C. 2017. Rhizosphere engineering: enhancing sustainable plant ecosystem productivity. Rhizosphere 3:233–243. doi:10.1016/j.rhisph.2017.04.012

[11] Chiaramonte JB, Mendes LW, Mendes R. 2021. Rhizosphere microbiome and soil-borne diseases, p 155–168. *In* Gupta VVSR, Sharma AK (ed), Rhizosphere biology: interactions between microbes and plants. Springer Singapore, Singapore. doi:10.1007/978-981-15-6125-2_7.

[12] Chen Q, Song Y, An Y, Lu Y, Zhong G. 2024. Soil microorganisms: their role in enhancing crop nutrition and health. Diversity (Basel) 16:734. doi:10.3390/d16120734

[13] Ou Y, Penton CR, Geisen S, Shen Z, Sun Y, Lv N, Wang B, Ruan Y, Xiong W, Li R, Shen Q. 2019. Deciphering underlying drivers of disease suppressiveness against pathogenic *Fusarium oxysporum*. Front Microbiol 10:2535. doi:10.3389/fmicb.2019.02535 PMC6861331 31781059

[14] Berendsen RL, Vismans G, Yu K, Song Y, de Jonge R, Burgman WP, Burmølle M, Herschend J, Bakker PAHM, Pieterse CMJ. 2018. Disease-induced assemblage of a plant-beneficial bacterial consortium. ISME J 12:1496–1507. doi:10.1038/s41396-018-0093-1 PMC5956071 29520025

[15] Russ D, Fitzpatrick CR, Teixeira PJPL, Dangl JL. 2023. Deep discovery informs difficult deployment in plant microbiome science. Cell 186:4496–4513. doi:10.1016/j.cell.2023.08.035 37832524

[16] Batista BD, Wang J, Liu H, Kaur S, Macdonald CA, Qiu Z, Trivedi P, Delgado-Baquerizo M, Xiong C, Liang J, Bange M, Singh BK. 2024. Biotic and abiotic responses to soilborne pathogens and environmental predictors of soil health. Soil Biol Biochem 189:109246. doi:10.1016/j.soilbio.2023.109246

[17] Wei F, Feng H, Zhang D, Feng Z, Zhao L, Zhang Y, Deakin G, Peng J, Zhu H, Xu X. 2021. Composition of rhizosphere microbial communities associated with healthy and *Verticillium* wilt diseased cotton plants. Front Microbiol 12:618169. doi:10.3389/fmicb.2021.618169 PMC8057349 33889135

[18] Wei Z, Hu J, Gu Y, Yin S, Xu Y, Jousset A, Shen Q, Friman V-P. 2018. *Ralstonia solanacearum* pathogen disrupts bacterial rhizosphere microbiome during an invasion. Soil Biol Biochem 118:8–17. doi:10.1016/j.soilbio.2017.11.012

[19] Schlüter S, Leuther F, Albrecht L, Hoeschen C, Kilian R, Surey R, Mikutta R, Kaiser K, Mueller CW, Vogel HJ. 2022. Microscale carbon distribution around pores and particulate organic matter varies with soil moisture regime. Nat Commun 13:2098. doi:10.1038/s41467-022-29605-w PMC9023478 35449155

[20] Wilpiszeski RL, Aufrecht JA, Retterer ST, Sullivan MB, Graham DE, Pierce EM, Zablocki OD, Palumbo AV, Elias DA. 2019. Soil aggregate microbial communities: towards understanding microbiome interactions at biologically relevant scales. Appl Environ Microbiol 85:e00324-19. doi:10.1128/AEM.00324-19 PMC6606860 31076430

[21] Fuentes B, Choque A, Gómez F, Alarcón J, Castro-Nallar E, Arenas F, Contreras D, Mörchen R, Amelung W, Knief C, Moradi G, Klumpp E, Saavedra CP, Prietzel J, Klysubun W, Remonsellez F, Bol R. 2021. Influence of physical-chemical soil parameters on microbiota composition and diversity in a deep hyperarid core of the Atacama Desert. Front Microbiol 12:794743. doi:10.3389/fmicb.2021.794743 PMC8859261 35197940

[22] Ren B, Hu Y, Chen B, Zhang Y, Thiele J, Shi R, Liu M, Bu R. 2018. Soil pH and plant diversity shape soil bacterial community structure in the active layer across the latitudinal gradients in continuous permafrost region of Northeastern China. Sci Rep 8:5619. doi:10.1038/s41598-018-24040-8 PMC5884794 29618759

[23] Yang B, Feng W, Zhou W, He K, Yang Z. 2024. Association between soil physicochemical properties and bacterial community structure in diverse forest ecosystems. Microorganisms 12:728. doi:10.3390/microorganisms12040728 PMC11052384 38674672

[24] Sagova-Mareckova M, Omelka M, Kopecky J. 2023. The golden goal of soil management: disease-suppressive soils. Phytopathology 113:741–752. doi:10.1094/PHYTO-09-22-0324-KD 36510361

[25] NOAA National Centers for Environmental Information. 2025. Climate at a glance: county time series. NOAA National Centers for Environmental Information. Available from: https://www.ncei.noaa.gov/access/monitoring/climate-at-a-glance/county/time-series

[26] Rashed A, Reed TD, Zeng Y, Wilkinson CA, Barts S. 2024. 2024 flue-cured tobacco production guide. Publ. Virginia Cooperative Extension, Blacksburg, VA. https://www.arec.vaes.vt.edu/2024-flue-cured-production-guide.

[27] United States Department of Agriculture. 2023. National organic program (NOP) standards. Agric Mark Serv. Available from: https://www.ams.usda.gov/rules-regulations/organic

[28] Zeng Y, Fulladolsa AC, Cordova AM, O’Neill P, Gray SM, Charkowski AO. 2020. Evaluation of effects of chemical soil treatments and potato cultivars on *Spongospora subterranea* soil inoculum and incidence of powdery scab and potato mop-top virus in potato. Plant Dis 104:2807–2816. doi:10.1094/PDIS-10-19-2202-RE 32954986

[29] R Core Team. 2025. R: a language and environment for statistical computing. R foundation for statistical computing. https://www.R-project.org/.

[30] Behrendt L, Larkum AWD, Trampe E, Norman A, Sørensen SJ, Kühl M. 2012. Microbial diversity of biofilm communities in microniches associated with the didemnid ascidian *Lissoclinum patella*. ISME J 6:1222–1237. doi:10.1038/ismej.2011.181 PMC3358027 22134643

[31] Yu Y, Lee C, Kim J, Hwang S. 2005. Group-specific primer and probe sets to detect methanogenic communities using quantitative real-time polymerase chain reaction. Biotechnol Bioeng 89:670–679. doi:10.1002/bit.20347 15696537

[32] Li R, Yang S, Lin M, Guo S, Han X, Ren M, Du L, Song Y, You Y, Zhan J, Huang W. 2022. The biogeography of fungal communities across different Chinese wine-producing regions associated with environmental factors and spontaneous fermentation performance. Front Microbiol 12:636639. doi:10.3389/fmicb.2021.636639 PMC8914289 35281311

[33] Li M, Inada M, Watanabe H, Suga H, Kageyama K. 2013. Simultaneous detection and quantification of *Phytophthora nicotianae* and *P. cactorum*, and distribution analyses in strawberry greenhouses by duplex real-time PCR. Microbes Environ 28:195–203. doi:10.1264/jsme2.me12177 PMC4070668 23614901

[34] Simko I, Piepho HP. 2012. The area under the disease progress stairs: calculation, advantage, and application. Phytopathology 102:381–389. doi:10.1094/PHYTO-07-11-0216 22122266

[35] Zeng Y, Abdo Z, Charkowski A, Stewart JE, Frost K. 2019. Responses of bacterial and fungal community structure to different rates of 1,3-dichloropropene fumigation. Phytobiomes J 3:212–223. doi:10.1094/PBIOMES-11-18-0055-R

[36] Caporaso JG, Kuczynski J, Stombaugh J, Bittinger K, Bushman FD, Costello EK, Fierer N, Peña AG, Goodrich JK, Gordon JI, et al.. 2010. QIIME allows analysis of high-throughput community sequencing data. Nat Methods 7:335–336. doi:10.1038/nmeth.f.303 PMC3156573 20383131

[37] Fasolo A, Deb S, Stevanato P, Concheri G, Squartini A. 2024. ASV vs OTUs clustering: effects on alpha, beta, and gamma diversities in microbiome metabarcoding studies. PLoS One 19:e0309065. doi:10.1371/journal.pone.0309065 PMC11449282 39361586

[38] McMurdie PJ, Holmes S. 2013. phyloseq: an R package for reproducible interactive analysis and graphics of microbiome census data. PLoS One 8:e61217. doi:10.1371/journal.pone.0061217 PMC3632530 23630581

[39] Oksanen J, Simpson GL, Blanchet FG, Kindt R, Legendre P, Minchin PR, Weedon J. 2001. vegan: community ecology package

[40] Wickham H, Averick M, Bryan J, Chang W, McGowan L, François R, Grolemund G, Hayes A, Henry L, Hester J, Kuhn M, Pedersen T, Miller E, Bache S, Müller K, Ooms J, Robinson D, Seidel D, Spinu V, Takahashi K, Vaughan D, Wilke C, Woo K, Yutani H. 2019. Welcome to the Tidyverse. J Open Source Softw 4:1686. doi:10.21105/joss.01686

[41] Gómez-Rubio V. 2017. ggplot2-elegant graphics for data analysis. J Stat Softw 77:1–3. doi:10.18637/jss.v077.b02

[42] Dawson C. 2021. ggprism: a “ggplot2” extension inspired by “GraphPad Prism”. CRAN: contributed packages.

[43] Wickham H, François R, Henry L, Müller K, Vaughan D. 2023. dplyr: a grammar of data manipulation

[44] Ma B, Wang H, Dsouza M, Lou J, He Y, Dai Z, Brookes PC, Xu J, Gilbert JA. 2016. Geographic patterns of co-occurrence network topological features for soil microbiota at continental scale in eastern China. ISME J 10:1891–1901. doi:10.1038/ismej.2015.261 PMC5029158 26771927

[45] Friedman J, Alm EJ. 2012. Inferring correlation networks from genomic survey data. PLoS Comput Biol 8:e1002687. doi:10.1371/journal.pcbi.1002687 PMC3447976 23028285

[46] Wickham H. 2007. Reshaping data with the reshape package. J Stat Softw 21:1–20. doi:10.18637/jss.v021.i12

[47] Cohen J. 2013. Statistical power analysis for the behavioral sciences. Routledge.

[48] Harrell Jr F. 2026. Hmisc: Harrell miscellaneous. R package version.

[49] Garg A, Kwakye S, Cates A, Peterson H, LaBine K, Olson G, Sharma V. 2025. Integrated soil health management influences soil properties: insights from a US Midwest study. Geoderma 455:117214. doi:10.1016/j.geoderma.2025.117214

[50] Grzyb A, Wolna-Maruwka A, Łukowiak R, Ceglarek J. 2022. Spatial and temporal variability of the microbiological and chemical properties of soils under wheat and oilseed rape cultivation. Agronomy 12:2259. doi:10.3390/agronomy12102259

[51] Gong B, He Y, Luo Z, Peng H, Cai H, Zhu Y, Bin J, Ding M. 2024. Response of rhizosphere soil physicochemical properties and microbial community structure to continuous cultivation of tobacco. Ann Microbiol 74:4. doi:10.1186/s13213-023-01748-1

[52] Mannaa M, Seo Y-S. 2021. Plants under the attack of allies: moving towards the plant pathobiome paradigm. Plants (Basel) 10:125. doi:10.3390/plants10010125 PMC7827841 33435275

[53] Orr R, Nelson PN. 2018. Impacts of soil abiotic attributes on *Fusarium* wilt, focusing on bananas. Appl Soil Ecol 132:20–33. doi:10.1016/j.apsoil.2018.06.019

[54] Otten W, Gilligan CA. 2006. Soil structure and soil‐borne diseases: using epidemiological concepts to scale from fungal spread to plant epidemics. Eur J Soil Sci 57:26–37. doi:10.1111/j.1365-2389.2006.00766.x

[55] Van De Graaf P, Lees AK, Wale SJ, Duncan JM. 2005. Effect of soil inoculum level and environmental factors on potato powdery scab caused by *Spongospora subterranea*. Plant Pathol 54:22–28. doi:10.1111/j.1365-3059.2005.01111.x

[56] Solís-García IA, Ceballos-Luna O, Cortazar-Murillo EM, Desgarennes D, Garay-Serrano E, Patiño-Conde V, Guevara-Avendaño E, Méndez-Bravo A, Reverchon F. 2020. *Phytophthora* root rot modifies the composition of the avocado rhizosphere microbiome and increases the abundance of opportunistic fungal pathogens. Front Microbiol 11:574110. doi:10.3389/fmicb.2020.574110 PMC7835518 33510714

[57] Feng Z, Liang Q, Yao Q, Bai Y, Zhu H. 2024. The role of the rhizobiome recruited by root exudates in plant disease resistance: current status and future directions. Environ Microbiome 19:91. doi:10.1186/s40793-024-00638-6 PMC11569615 39550594

[58] Gu Y, Wei Z, Wang X, Friman V-P, Huang J, Wang X, Mei X, Xu Y, Shen Q, Jousset A. 2016. Pathogen invasion indirectly changes the composition of soil microbiome via shifts in root exudation profile. Biol Fertil Soils 52:997–1005. doi:10.1007/s00374-016-1136-2

[59] Kumar V. 2014. Optimal physical parameters for growth of *Trichoderma* species at varying pH, temperature and agitation. Virol Mycol 03:127. doi:10.4172/2161-0517.1000127

[60] Gangwar P, Erickson LJ, Barts S, Johnson CS, Reed TD, Zeng Y. 2026. Identification and pathogenicity of *Fusarium* species associated with *Fusarium* wilt of flue-cured tobacco. Plant Dis 110:79–88. doi:10.1094/PDIS-12-24-2715-SR 40313001

[61] Handoo ZA, Subbotin SA. 2018. Taxonomy, identification and principal species, p 365–398. *In* Perry RN, Moens M, Jones JT (ed), Cyst nematodes. CAB International, Wallingford, UK.

[62] Yang H, Li J, Xiao Y, Gu Y, Liu H, Liang Y, Liu X, Hu J, Meng D, Yin H. 2017. An integrated insight into the relationship between soil microbial community and tobacco bacterial wilt disease. Front Microbiol 8:2179. doi:10.3389/fmicb.2017.02179 PMC5681905 29163453

[63] Miljaković D, Marinković J, Balešević-Tubić S. 2020. The significance of *Bacillus* spp. in disease suppression and growth promotion of field and vegetable crops. Microorganisms 8:1037. doi:10.3390/microorganisms8071037 PMC7409232 32668676

[64] Dobrzyński J, Naziębło A. 2024. *Paenibacillus* as a biocontrol agent for fungal phytopathogens: is *P. polymyxa* the only one worth attention? Microb Ecol 87:134. doi:10.1007/s00248-024-02450-8 PMC11527970 39480531

[65] Xia X, Wei Q, Wu H, Chen X, Xiao C, Ye Y, Liu C, Yu H, Guo Y, Sun W, Liu W. 2024. *Bacillus* species are core microbiota of resistant maize cultivars that induce host metabolic defense against corn stalk rot. Microbiome 12:156. doi:10.1186/s40168-024-01887-w PMC11342587 39180084

[66] Mehmood N, Saeed M, Zafarullah S, Hyder S, Rizvi ZF, Gondal AS, Jamil N, Iqbal R, Ali B, Ercisli S, Kupe M. 2023. Multifaceted impacts of plant-beneficial *Pseudomonas* spp. in managing various plant diseases and crop yield improvement. ACS Omega 8:22296–22315. doi:10.1021/acsomega.3c00870 PMC10308577 37396244

[67] Liu Y, Gates AD, Liu Z, Duque Q, Schmidt SS, Chen MY, Hamilton CD, O’Toole GA, Haney CH. 2026. *In vitro* biofilm formation by a beneficial bacterium partially predicts *in planta* protection against rhizosphere pathogens. ISME J 20. doi:10.1093/ismejo/wraf114 PMC12919676 40454553

[68] Ebrahimi-Zarandi M, Saberi Riseh R, Tarkka MT. 2022. Actinobacteria as effective biocontrol agents against plant pathogens, an overview on their role in eliciting plant defense. Microorganisms 10:1739. doi:10.3390/microorganisms10091739 PMC9500821 36144341

