# Supplementary material for: Reduced soil microbial diversity and altered co-occurrence networks associated with Phytophthora nicotianae infection in flue-cured tobacco fields
Source: mSphere. 2026 Aug 31;11(9):e00501-26. doi: 10.1128/msphere.00501-26 (PMC13621835; doi:10.1128/msphere.00501-26)
Supplement: Supplementary file 1 [file msphere.00501-26-s0001.pdf]

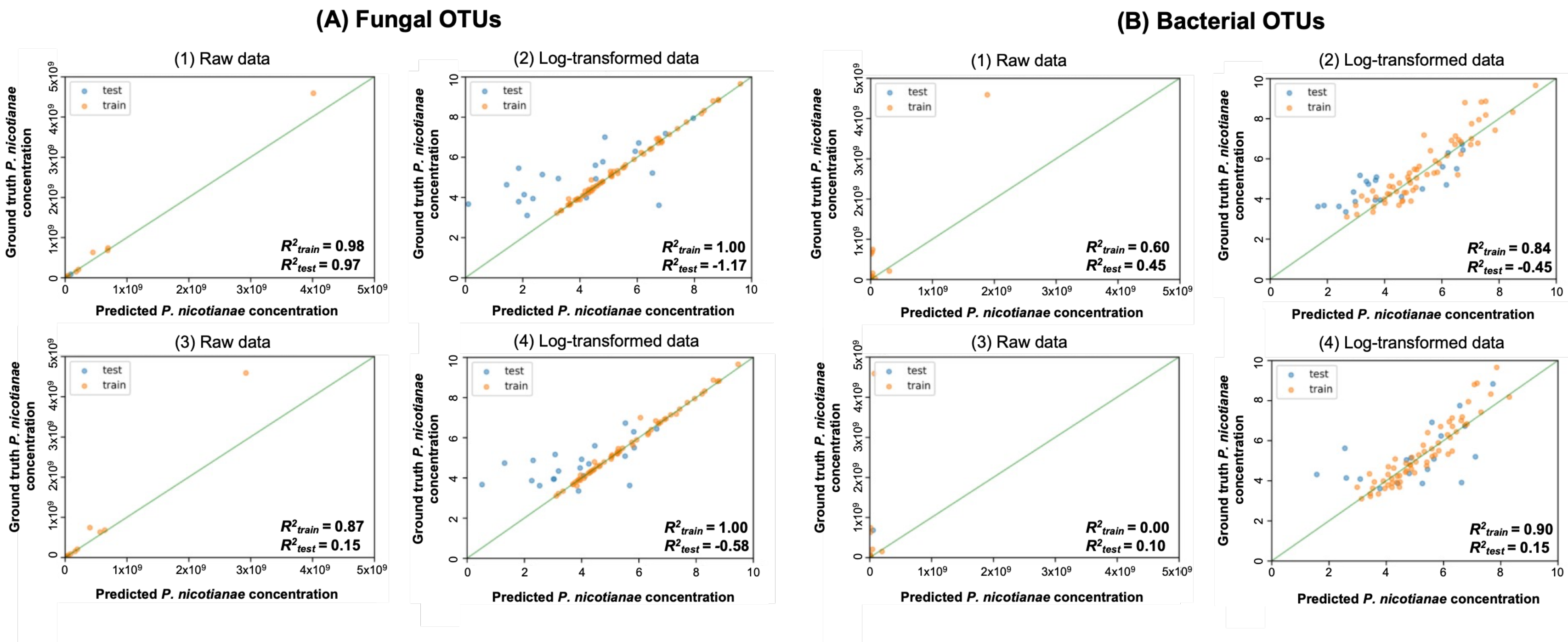

Fig. S1. Regression performance of XGBoost models predicting *P. nicotianae* DNA concentration using (A) fungal and (B) bacterial OTUs under raw and log-transformed scales, evaluated across two train-test splits. For each data scale (raw or log-transformed), models were assessed using two independent train-test splits with different random seeds (Split set 1: panels 1-2; Split set 2: panels 3-4).

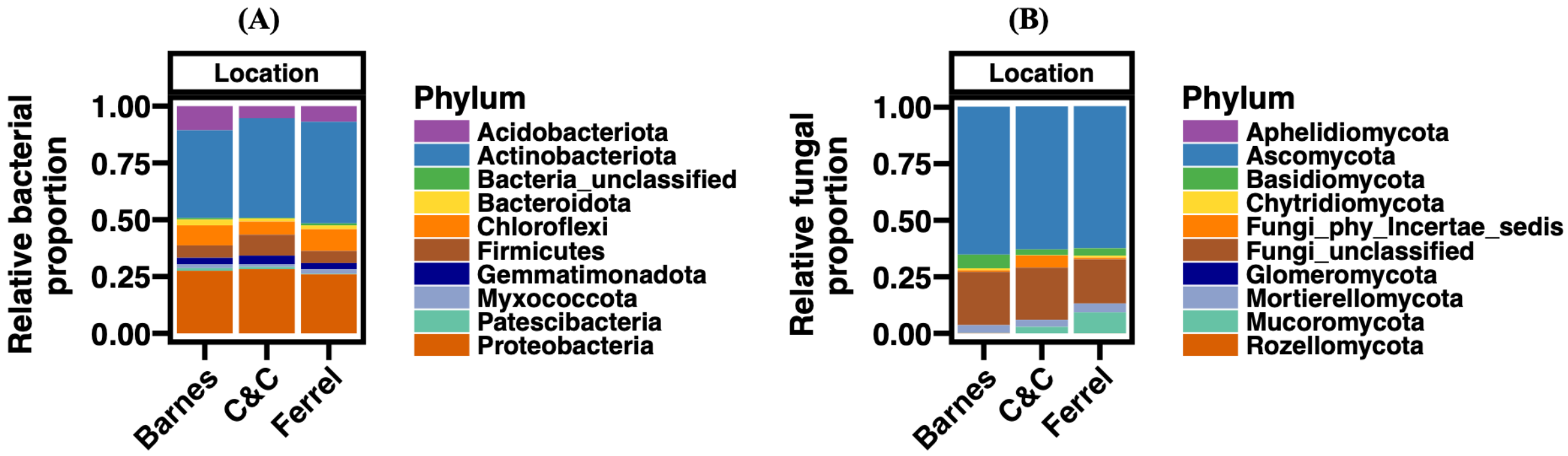

Fig. S2. The relative proportion of the top 10 bacterial (A) and fungal (B) taxa at the phylum level at three tobacco farms (P-values$_{\text{PERMANOVA}} > 0.05$).

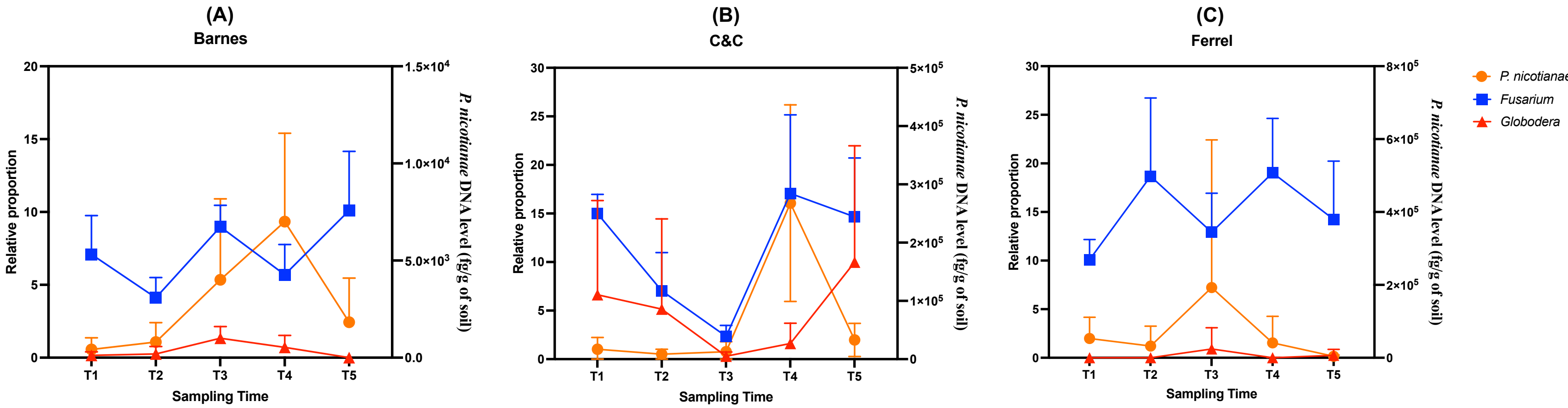

Fig. S3. Temporal patterns of *Fusarium* genus, *Globodera* genus, and *P. nicotianae* across sampling times (T1-T5) in (A) Barnes, (B) C&C, and (C) Ferrel fields. For the T3-T4 interval in C&C, the relationship between *Fusarium* and *P. nicotianae* was significant (R^2 = 0.6632; P = 0.0366), whereas the relationship between *Globodera* and *P. nicotianae* was not (R^2 = 0.0876; P = 0.8098).
